# Chronic low back pain patient groups in primary care – A cross sectional cluster analysis

**DOI:** 10.1186/1471-2474-14-294

**Published:** 2013-10-16

**Authors:** Annika Viniol, Nikita Jegan, Oliver Hirsch, Corinna Leonhardt, Markus Brugger, Konstantin Strauch, Jürgen Barth, Erika Baum, Annette Becker

**Affiliations:** 1 Department of General Practice / Family Medicine, Philipps University of Marburg, Karl-von-Frisch-Str. 4, 35043 Marburg, Germany; 2 Institute of Medical Informatics, Biometry and Epidemiology, Chair of Genetic Epidemiology, Ludwig Maximilians University, Munich, Ingolstädter Landstraße 1, 85764 Neuherberg, Germany; 3 Institute of Genetic Epidemiology, Helmholtz Center Munich – German Research Center for Environmental Health, Ingolstädter Landstraße 1, 85764 Neuherberg, Germany; 4 Institute for Social and Preventive Medicine (ISPM), University of Bern, Niesenweg 6, 3012 Bern, Switzerland

**Keywords:** Subgroups, Chronic low back pain, Primary care

## Abstract

**Background:**

Due to the heterogeneous nature of chronic low back pain (CLBP), it is necessary to identify patient groups and evaluate treatments within these groups. We aimed to identify groups of patients with CLBP in the primary care setting.

**Methods:**

We performed a k-means cluster analysis on a large data set (n = 634) of primary care patients with CLBP. Variables of sociodemographic data, pain characteristics, psychological status (i.e., depression, anxiety, somatization), and the patient resources of resilience and coping strategies were included.

**Results:**

We found three clusters that can be characterized as “pensioners with age-associated pain caused by degenerative diseases”, “middle-aged patients with high mental distress and poor coping resources”, and “middle-aged patients who are less pain-affected and better positioned with regard to their mental health”.

**Conclusions:**

Our results supported current knowledge concerning groups of CLBP patients in primary care. In particular, we identified a group that was most disabled and distressed, and which was mainly characterized by psychological variables. As shown in our study, pain-related coping strategies and resilience were low in these patients and might be addressed in differentiating treatment strategies. Future studies should focus on the identification of this group in order to achieve effective treatment allocation.

**Trial registration:**

German Clinical Trial Register DRKS00003123

## Background

Chronic pain syndromes, especially chronic low back pain (CLBP) is one of the most prevalent diseases in Western populations [1] and is one of the most common reasons for primary care consultations [2]. CLBP is associated with a high burden of disease with respect to health care costs and patients’ quality of life [3].

Current treatment recommendations emphasize a multidisciplinary approach for chronic lower back pain (CLBP) treatment [4]. This therapy approach is effective, but often unrealistic as it is organizationally challenging, expensive, and cannot be made accessible to all CLBP patients [5].

A possible solution could be considering whether every CLBP patient needs the multidisciplinary treatment. Considering the heterogeneous and multifactorial nature of chronic pain patients, it might be necessary to differentiate the main characteristics of patient groups and evaluate individual treatment approaches [6].

Several attempts have been made to identify groups of pain patients. Studies are typically based on single dimensions such as traditional psychological measures (e.g., the Minnesota Multiphasic Personality Inventory or the Symptom Checklist-90-R) [7], pain expression [8], physical performance [9], patient perceived etiology [10], and psychophysiological responses [11]. Multifactorial analyses usually study the impact of psychosocial characteristics in conjunction with pain-related fear [12,13], emotional distress [14,15], behavioral response [16,17], or other psychosocial and behavioral characteristics [18]. These studies are driven by prognostic risk factors; none focuses on protective factors like coping strategies and self-efficacy, which influence chronic pain in a positive way.

We aimed to identify groups of patients with CLBP in the primary care setting. For group analyses, we considered pain characteristics, common psychological characteristics (i.e., depression, anxiety, somatization), and also included patient resources like resilience and coping strategies, which might contribute to future recommendations for CLBP patient counseling.

## Methods

### Study design

Our data referred to the cross sectional baseline analysis of a 12-month cohort study that identified risk factors and protective factors of pain generalization in primary care CLBP patients. A detailed study protocol has been published elsewhere [19].

This project is part of the research consortium LOGIN “Localized and Generalized Musculoskeletal Pain: Psychobiological Mechanisms and Implications for Treatment”, funded by the German Federal Ministry of Education and Research.

### Study population

During a 5-month period, fifty-eight general practitioners (evenly distributed in the northern region of Hessen in Germany) consecutively enrolled all eligible patients consulting for CLBP as a primary or secondary consulting reason (inclusion criteria). The symptom “chronic low back pain” was defined as back pain below the costal margin and above the inferior gluteal folds (with or without pain radiation), which had started at least three months prior and continued during most days (i.e., more than 50%) in the last three months. Patients under 18 years, pregnant women, and persons with an insufficient understanding of the German language or severe cognitive impairments (e.g., dementia) were excluded from the study.

Patients who gave their informed consent were asked to complete a questionnaire directly after the consultation or at home. During the recruitment period, trained clinical monitors conducted two random quality control audits of the GPs’ performance.

### Measurements

To explore descriptive characteristics, the questionnaire included the following physical and psychological parameters (for detailed information please see Viniol et al. [19]).

#### ***Pain characteristics and sociodemographic data***

To evaluate the number of different pain areas, we measured pain localization with the “body pain drawing model” proposed by Pfau et al. [20]. Pain anamnesis and sociodemographic data were determined with the “German Pain Questionnaire”, the official pain questionnaire of the German Association for the Study of Pain [21]. We used the following DGSS modules: duration, characteristics, course of pain, sociodemographic data, health care utilization, and medication.

We used the three-item social support subscale from the West Haven-Yale Multidimensional Pain Inventory (WHYMPI) to explore the partner’s reaction in response to patient’s pain (internal consistency of the subscales: α = 0.63-0.90) [22,23].

The severity of chronic pain was measured by the German translation of von Korff’s Graded Chronic Pain questionnaire (GCP) [24]. Severity is computed from “pain intensity” and “pain-related disability” (internal consistency of subscales: α = 0.68-0.88) [25].

#### ***Comorbidities***

Using the Self-Administered Comorbidity Questionnaire (SACQ), we asked the patients about 14 common medical conditions: high blood pressure, heart disease, asthma, chronic obstructive pulmonary disease, ulcer/stomach disease, diabetes, high blood lipid level, kidney disease, osteoarthritis/degenerative arthritis, rheumatoid arthritis, osteoporosis, cancer disease, depression, other psychiatric diseases [26,27].

#### ***Psychological parameters and patient resources***

Psychosomatic symptoms were measured with the somatization subscale of the Symptom Checklist 90-Revised (SCL-90-R), a commonly used psychological status symptom inventory for psychopathology (internal consistency: α = 0.81) [28].

Screening for anxiety disorders and depression was done by the Hospital Anxiety and Depression Scale (HADS) (internal consistency anxiety α = 0.80; depression α = 0.81) [29,30].

Coping resources for back pain were evaluated by the FBR (*Fragebogen zu Bewältigungsressourcen bei Rückenschmerzen*) questionnaire from Tamcam et al. [31].

We used the resilience scale RS-11, a shortened and validated German form of the Wagnild & Young questionnaire, to assess the resilience (internal consistency α = 0.91) [32,33].

### Statistical analyses

We performed k-means cluster analyses generalized to all scales of measurement with squared euclidean distances [34]. The k-means procedure identifies relatively homogenous groups while maximizing the variability between clusters. Variables with mixed scaling can be handled in cluster analysis [34,35]. Calculations were done with ALMO 15 (http://www.almo-statistik.de), which includes a k-means algorithm that is able to handle the different scalings of our variables and the large sample size. This program provides statistical measures to evaluate the appropriateness of a cluster solution (F-value, eta^2^) [35].

Cluster analysis is an iterative process looking for the most relevant variables adding to an interpretable solution [36,37]. Therefore, we ran several analyses for selection of variables, based on a variable-specific eta^2^ < 0.05. Accordingly, the following variables were excluded: gender, living with a partner, applied for pension, pain distribution, medication, WHYMPI – social support scale, education level, kind of job, time of pain. We attached special importance to certain individual variables (number of pain areas, therapeutic strategies, consultations, operations) so that they were included irrespective of their eta^2^.

### Ethics statement

The study was approved by the local ethics commission of Philipps University of Marburg, Germany (Ethik: 11.06.2010, AZ 88/10) and is in accordance with the Declaration of Helsinki.

## Results

### Recruitment data

Fifty-eight GPs with various practice characteristics (i.e., size, locations, and organizational structures) participated in the study. During the five-month recruitment period, they identified 746 eligible patients. Of these, 655 patients agreed to participate. A total of 647 subjects were analyzed; 8 patients were excluded because they did not report lower back pain in the pain drawing.

### Total group characteristics

On average, the participating CLBP patients were 56 years old (SD 14.0; age range: 20-88 years). The majority was female, married, and living in a two-person household. Just over half (52.4%) reported having back pain for more than ten years. Half of the participants were employed; 74.6% of the unemployed participants were retired. Table 1 shows the characteristics of all recruited CLBP patients.

**Table 1:** Sociodemographic data of the analyzed patients (n = 634)

**Variable**	
**Sex** [no. (%)] n = 634	
Female	388 (61.2)
**Age** [mean: years (SD)] n = 634	56.30 (13.95)
**Nationality** [no. (%)] n = 619	
German	610 (98.5)
Other	9 (1.5)
**Marital status** [no. (%)] n = 634	
Single	82 (12.9)
Married	415 (65.5)
Divorced	80 (12.6)
Widowed	57 (9.0)
**Living with a partner** [no. (%)] n = 611	477 (78.1)
**Persons at household** [no. (%)] n = 605	
1 person	87 (14.4)
2 persons	297 (49.1)
3 persons	114 (18.8)
4 persons	74 (12.2)
> 4 persons	33 (5.4)
**Level and years of education** [no. (%)] n = 632	
13/12 years	100 (15.8)
10 years	191 (30.2)
9 years	322 (50.9)
Other graduation	15 (2.4)
No qualification	4 (0.6)
**Employment status** [no. (%)] n = 631	
Working (full or part-time)	322 (51.0)
**Reasons for not working** [no. (%)] n = 306	
Keeping house	48 (15.7)
Retired	228 (74.6)
Unemployed	27 (8.8)
Other	3 (1.0)
**First time of back pain** [no (%)] n = 634	
Since < 1 year	77 (12.2)
Since 1-2 years	47 (7.4)
Since 2-5 years	86 (13.6)
Since 5-10 years	92 (14.5)
Since > 10 years	332 (52.4)

### Description of cluster modeling

A three-cluster solution resulted in an F_max_-value of 76.16 and an eta^2^ of 0.191, meaning that 19.1% of the variance can be explained by this partitioning and can be considered as a cluster solution with acceptable quality criteria [34,37,38].

The variables used for classification are depicted in Table 2. All included variables contributed substantially to the breakdown into the three clusters. Highest variable-specific eta^2^ values for the most important variables for cluster partitioning were found for employment status (eta^2^ = 0.44), days of sick leave (eta^2^ = 0.44), and age (eta^2^ = 0.40). In addition, having a hobby/enjoyment as a coping resource (eta^2^ = 0.41), depression (eta^2^ = 0.31), retirement status (eta^2^ = 0.35), and resilience (eta^2^ = 0.28) were also found to play an important role in cluster partitioning. All variables used in the cluster analysis are listed in Table 2.

**Table 2:** Contribution of classification variables to the separation of cluster (n = 634)

**Variable**	**F**_**max**_**-value**	**Variable specific eta**^**2**^
**Age**	208.40	0.40
**Number of persons at household**	39.18	0.12
**Employment status**		
- Working (full or part-time)	242.31	0.44
**Reasons for not working**		
- Keeping house	31.37	0.17
- Unemployed	16.61	0.10
- Retired	81.44	0.35
- Other	5.38	0.03
**Days on sick leave**	117.13	0.44
**Degree of disability**		
- Yes	56.83	0.16
**Number of pain areas**	4.84	0.02
**Index of the graded chronic pain (von Korff)**		
- 0	-	-
- 1	4.33	0.02
- 2	18.85	0.06
- 3	0.43	0.00
- 4	25.73	0.08
**Gut feeling**: **Will the pain get away?**		
- Yes	33.29	0.10
**Number of different therapeutic strategies**	19.81	0.06
**Number of consultations because of back pain/6 months**	12.14	0.04
**Number of operations because of back pain**	14.33	0.05
**Symptom check-list-90-R (Somatization)**	41.18	0.13
**Self-administered comorbidity questionnaire (SACQ)**		
- Psychological comorbidities*	23.87	0.09
- Musculoskeletal comorbidities **	56.47	0.19
- Other comorbidities ***	30.61	0.11
**Brief resilience scale (RS-11)**	118.04	0.28
**Hospital anxiety and depression (HADS)**		
- Anxiety	67.69	0.11
- Depression	141.60	0.31
**Coping resources of back pain (FBR)**		
- Emotional social support	112.55	0.26
- Practical help	85.62	0.22
- Exercise and relaxation	101.90	0.25
- Hobby and enjoyment	215.64	0.41
- Cognitive strategies	183.63	0.37
- Knowledge	86.56	0.22
- Spirituality	63.15	0.17

*Participants with the comorbidities depression and/or other psychiatric diseases.

**Participants with the comorbidities degenerative arthritis and/or rheumatoid arthritis and/or osteoporosis.

***Participants with the comorbidities high blood pressure and/or heart diseases and/or asthma and/or chronic obstructive pulmonary disease and/or ulcer/stomach diseases and/or diabetes and/or high blood lipid level and/or kidney diseases and/or cancer diseases.

### Description of clusters

Table 3 depicts the description of the three clusters by means and proportions of classification variables.

**Table 3:** Characterization of the three clusters of CLBP patients (n = 634)

	**Cluster 1**	**Cluster 2**	**Cluster 3**
	n = 179	n = 200	n = 255
	(28.2%)	(31.5%)	(40.2%)
**Age** [years: mean (SD)]	68.2 (8.6)	57.8 (13.1)	46.8 (10.3)
**Persons at household** [mean (SD)]	2.1 (0.9)	2.2 (0.1)	2.9 (1.2)
**Employment status** [no. (%)]			
- Working (full or part-time)	12 (6.8)	88 (44.2)	222 (87.1)
**Reasons for not working** [no. (%)]			
- Keeping house	11 (6.7)	20 (17.7)	19 (57.9)
- Unemployed#	6 (3.7)	10 (8.8)	11 (33.3)
- Retired	146 (89.6)	82 (72.6)	1 (3.0)
- Other	0 (0)	1 (0.9)	2 (6.1)
**Days on sick leave of the employed participants** [mean (SD)]	81.4 (14.4)	20.9 (26.7)	4.8 (8.2)
**Degree of disability** [no. (%)]			
- Yes	96 (58.2)	113 (59.2)	46 (18.6)
**Number of pain areas** [mean (SD)]	3.9 (2.3)	4.4 (2.2)	3.7 (2.2)
**Index of the graded chronic pain (von Korff)** [no (%)]			
- 0	0 (0)	0 (0)	0 (0)
- 1	23 (14.5)	15 (8.1)	44 (18.0)
- 2	32 (20.1)	24 (13.0)	91 (37.1)
- 3	51 (32.1)	58 (31.4)	69 (28.2)
- 4	53 (33.3)	88 (47.6)	41 (16.7)
**Symptom check-list-90-R (Somatization)** [mean (SD)]			
	11.1 (6.9)	14.4 (7.2)	8.8 (5.0)
**Self-administered comorbidity questionnaire** (SACQ) [no (%)]			
- Psychological comorbidities*	23 (19.5)	67 (44.4)	35 (15.2)
- Musculoskeletal comorbidities**	89 (75.4)	87 (57.6)	56 (24.3)
- Other comorbidities***	107 (90.7)	117 (77.5)	125 (54.3)
**Brief resilience scale** (RS-11) [mean (SD)]	61.3 (11.9)	47.3 (13.6)	63.2 (8.7)
**Hospital anxiety and depression** (HADS) [mean (SD)]			
- Anxiety	6.6 (3.4)	10.5 (4.1)	7.0 (3.3)
- Depression	7.4 (2.6)	10.9 (2.9)	6.8 (2.4)
**Coping resources of back pain (FBR)** [mean (SD)]			
- Emotional social support	13.9 (5.0)	6.5 (4.6)	11.6 (5.2)
- Practical help	13.5 (5.5)	7.5 (4.8)	13.2 (5.1)
- Exercise and relaxation	14.2 (4.4)	8.2 (4.2)	13.1 (4.7)
- Hobby and enjoyment	16.0 (3.6)	8.0 (4.4)	14.7 (4.0)
- Cognitive strategies	15.7 (3.9)	7.8 (4.3)	14.1 (4.5)
- Knowledge	7.0 (2.6)	4.1 (2.5)	6.9 (2.4)
- Spirituality	6.2 (3.3)	2.5 (2.8)	3.8 (3.5)
**Gut feeling**: **Will the pain get away?** [no (%)]			
- Yes	90 (50.3)	73 (36.5)	40 (15.7)
**Number of different therapeutic strategies** [mean (SD)]	7.1 (3.8)	6.6 (3.9)	5.0 (3.4)
**Number of consultations because of back pain / 6 months** [mean (SD)]	5.7 (5.9)	6.1 (7.2)	3.6 (4.2)
**Number of operations because of back pain** [mean (SD)]	0.3 (0.8)	0.5 (1.4)	0.1 (0.3)

*Participants with the comorbidities depression and/or other psychiatric diseases.

**Participants with the comorbidities degenerative arthritis and/or rheumatoid arthritis and/or osteoporosis.

***Participants with the comorbidities high blood pressure and/or heart diseases and/or asthma and/or chronic obstructive pulmonary disease and/or ulcer/stomach diseases and/or diabetes and/or high blood lipid level and/or kidney diseases and/or cancer diseases.

#Proportion of unemployment among persons who are not working and are in an employable age (≤ 65 years): Cluster 1: 15.4%; Cluster 2: 20.0%; Cluster 3: 36.7%.

#### ***Cluster 1 - “pensioners with age-associated pain caused by degenerative diseases”***

(n = 179, 28.2% of all patients) comprised mainly retired persons with an average age of 68.2 years (SD 8.6; age range: 42-88 years). Patients in this cluster suffered from a medium pain severity. In most cases, they had musculoskeletal and other chronic cardiovascular/pulmonary/stomach diseases. In comparison to the other cluster groups, cluster 1 scored highest regarding perceived efficacy of coping resources and mental disorders. Furthermore, patients in cluster 1 were optimistic with regard to their pain prognoses.

#### ***Cluster 2 - “middle-aged patients with high mental distress and poor coping resources”***

(n = 200, 31.5% of all patients) consisted of middle-aged patients (mean 57.8 years, SD 13.1; age range: 26-87 years) with only 44.2% employed. Most unemployed patients were pensioners due to disability (72.6%). Remaining unemployed patients were either keeping house (17.7%) or seeking work (8.8%). Patients from cluster 2 had the most pronounced pain (highest rate of high pain severity and number of pain areas) and a high rate of pain-related disability. In comparison to the other clusters, patients in this cluster had more psychological distress (highest rate of the psychological comorbidities anxiety, depression, and symptoms of somatization). Furthermore, they had the worst resilience and the least efficient coping resources. They were high health care utilizers regarding the number of consultations and operations. We called them “middle-aged patients with high mental distress and poor coping resources”.

#### ***Cluster 3 - “middle-aged patients who are less pain-affected and better positioned with regard to their mental health”***

(n = 255, 40.2% of all patients) was also comprised of middle-aged (mean 46.8 years; SD 10.3; age range: 20-73years), but mostly working (87.1%) patients. In comparison to the second cluster, they were less pain affected, had fewer comorbidities and showed a better mental health (lower rates of anxiety, depression, and psychological comorbidities). Resilience was higher and coping resources were more pronounced than in patients belonging to cluster 2. In addition, they rarely required health care.

Table 4 shows a summary of the main cluster characteristics.

**Table 4:** Main characteristics of the three clusters

	**Cluster 1**	**Cluster 2**	**Cluster 3**
**Age on average**	68	58	47
**Working status of the majority**	Pensioners	Invalidity pensioners	Working
**Severity of pain**	Intermediate	High	Low
**Mental disorders** (somatization, anxiety, depression, psychological comorbidities*)	Intermediate	High	Low
Musculoskeletal**/other***comorbidities	High	Low	Intermediate
**Resources (resilience, coping)**	Intermediate	Low	High
**Health care utilization**	Intermediate	High	Low

*Participants with the comorbidities depression and/or other psychiatric diseases.

**Participants with the comorbidities degenerative arthritis and/or rheumatoid arthritis and/or osteoporosis.

***Participants with the comorbidities high blood pressure and/or heart diseases and/or asthma and/or chronic obstructive pulmonary disease and/or ulcer/stomach diseases and/or diabetes and/or high blood lipid level and/or kidney diseases and/or cancer diseases.

## Discussion

We performed a k-means cluster analysis on a large data set of primary care patients with chronic low back pain. We found three clusters that can be characterized as “pensioners with age-associated pain caused by degenerative diseases”, “middle-aged patients with high mental distress and poor coping resources”, and “middle-aged patients who are less pain-affected and better positioned with regard to their mental health”.

Several researchers have stated the need to identify patient groups that could serve as target groups for effective treatment strategies [6]. Turk et al. identified three groups of chronic pain patients by the WHYMPI [22,39]. The first group, “dysfunctional patients”, corresponds to patients with high pain severity, a low activity level, marked interference with everyday life due to pain, high affective distress, and low perception of life control. The second group, “adaptive copers”, is characterized by a lower pain severity, a higher activity level, lower interference and affective distress, and higher life control. The third group, “interpersonally distressed”, features middle pain severity, general activity, interference and affective distress, and lower social support than the other two groups.

Shaw et al. identified four groups of patients with acute work-related back pain based on disability risk factors (pain, depressive mood, fear avoidance beliefs, work inflexibility, and poor expectations for recovery) [40]. Group one consists of patients who are most affected by pain, concerned with high physical demands at work. This group resembles the “fear avoidance” category and shows low expectations of returning to work. Group two is characterized by a high rate of emotional distress and above average pain intensity. Patients in group three are identified by a high degree of concern about job placement. Finally, patients from group four show low risk factors for disability. They have positive expectations for workplace accommodation and returning to normal work.

Boersma et al. identified groups of acute and subacute spinal pain patients with regard to their risk for permanent pain or disability [41]. Their group profiles “fear-avoidant”, “distressed fear-avoidant”, “low risk” and “low risk depressed mood” are comparable to the results of Shaw et al.

Even though the included variables and patient populations (e.g., acute vs. chronic pain, low vs. no low back pain, different settings) of the aforementioned studies differ, they all have one aspect in common: All analyses revealed one patient group which seems most distressed and shows above average mental distress and high pain severity. In this way, mental health status seems to be a key differential factor.

The primary care setting comprises a high prevalence of older, often multimorbid patients and many chronic diseases. Therefore, groups identified in different settings might not be relevant for general practice [42]. Even though we could confirm the presence of middle-aged groups with minor and major psychosocial distress, further studies are required; diagnostic studies to identify these groups and treatment studies, which would prove effectiveness of group-specific treatments.

Hill et al. developed the STarT Back tool for the primary care setting [43]. The tool classifies patients with low back pain (LBP) into three groups based on nine questions referring to potentially modifiable physical and psychological prognostic indicators for persistent, disabling symptoms. Patients are categorized as “low risk”, “medium risk”, and “high risk” for future disabling LBP. However, the studies from Hill et al. included patients with acute and chronic LBP. Our study focused only on patients with chronic LBP.

### Limitations

Our study is subject to selection bias. GPs may have subconsciously preferred to recruit special cases (e.g., patients with higher disease severity or special personality), or forgotten to recruit patients due to high workload. Furthermore, some patients may have refused to participate in our study due to the long questionnaire, especially considering that our study population included a large proportion of older people with age-associated mental deficiencies. These factors might reduce the external validity of our results. In general, a limitation of cluster analysis is that the results depend on the input variables [44].

Since the less pain-affected patients of cluster three (average age: 46.8 years) are younger than the more pain-affected patients of cluster two (average age: 57.8 years), it is possible that younger patients from the third cluster will move to the second cluster as they age. This is especially likely because increased age is a proven risk factor for increased pain outcomes (e.g., transition from localized to widespread pain) [45]. We should soon be able to prove this hypothesis using follow-up data from our cohort study.

## Conclusion

Our results supported current knowledge concerning groups of CLBP patients in primary care. This knowledge could be the starting point for developing a group-specific therapy for general practitioners.

For the group “pensioners with age-associated pain caused by degenerative diseases”, a therapeutic orientation aligned with the guideline “pain of older people” [46,47] would be appropriate. It involves evidence-based treatment approaches individually adapted to older people and their comorbidities.

The group “patients of an employable age with high mental distress and poor coping resources” should receive multimodal pain therapy with a particular focus on psychotherapy to improve coping resources and resilience. With regard to the fact that mental disorders, especially depression, are the most decisive cost factor of chronic pain [48], it is particularly important to treat them in an effective manner.

For the group “patients of an employable age who are less pain-affected and better positioned with regards to their mental conditions”, a general practitioner-based, time-contingent monitoring should be indicated. It should be planned independent of pain exacerbations and should be conducted according to the bio-psycho-social model of pain.

Next step of our research will be to develop a therapy study which evaluates the efficacy of group-specific therapy approaches.

## Abbreviations

CLBP: Chronic low back pain; GP: General practitioner; WHYMPI: West haven-uyale multidimensional pain inventory; GCP: Graded chronic pain; SACQ: Self-administered comorbidity questionnaire; SCL-90-R: Symptom checklist 90-revised; HADS: Hospital anxiety and depression scale; FBR: *Fragebogen zu Bewältigungsressourcen bei Rückenschmerzen* (questionnaire for assessing coping resources for back pain); RS: Resilience.

## Competing interests

Annika Viniol does not state any financial or non-financial conflicts of interest.

Nikita Jegan does not state any financial or non-financial conflicts of interest.

Oliver Hirsch does not state any financial or non-financial conflicts of interest.

Corinna Leonhardt does not state any financial or non-financial conflicts of interest.

Markus Brugger does not state any financial or non-financial conflicts of interest.

Konstantin Strauch does not state any financial or non-financial conflicts of interest.

Jürgen Barth does not state any financial or non-financial conflicts of interest.

Erika Baum does not state any financial or non-financial conflicts of interest.

Annette Becker was a consultant for Grünenthal GmbH, from whom she has also received a speaker’s fee.

## Authors’ contributions

AV planned the study, collected and analyzed data and wrote the manuscript. NJ collected and analyzed data. OH analyzed data. MB and KS gave statistical support and planned the study. JB and EB planned and revised study design. CL and AB planned the study, discussed the results and revised the manuscript critically. All authors edited the drafted version of the manuscript. All authors read and approved the final manuscript.

## Pre-publication history

The pre-publication history for this paper can be accessed here:

http://www.biomedcentral.com/1471-2474/14/294/prepub
